# Possible Emergence of West Caucasian Bat Virus in Africa

**DOI:** 10.3201/eid1412.080750

**Published:** 2008-12

**Authors:** Ivan V. Kuzmin, Michael Niezgoda, Richard Franka, Bernard Agwanda, Wanda Markotter, Janet C. Beagley, Olga Yu Urazova, Robert F. Breiman, Charles E. Rupprecht

**Affiliations:** Centers for Disease Control and Prevention, Atlanta, Georgia, USA (I.V. Kuzmin, M. Niezgoda, R. Franka, O.Y. Urazova, C.E. Rupprecht); National Museum of Kenya, Nairobi, Kenya (B. Agwanda); University of Pretoria, Pretoria, South Africa (W. Markotter); University of Georgia, Athens, Georgia, USA (J.C. Beagley); Centers for Disease Control and Prevention Kenya, Nairobi (R.F. Breiman)

**Keywords:** West Caucasian bat virus, lyssavirus, bats, Miniopterus, seroprevalence, dispatch

## Abstract

The prevalence of neutralizing antibody against West Caucasian bat virus (WCBV) in *Miniopterus* bats collected in Kenya ranged from 17% to 26%. Seropositive bats were detected in 4 of 5 locations sampled across the country. These findings provide evidence that WCBV, originally isolated in Europe, may emerge in other continents.

Bats are reservoir hosts of several emerging zoonotic RNA viruses (*1*). In particular, bats host a range of lyssaviruses, as has been reported from different continents (*2*). Presently, 7 species are recognized within the genus *Lyssavirus* (order Mononegavirales, family *Rhabdoviridae*): *Rabies virus* (RABV), *Lagos bat virus* (LBV), *Mokola virus* (MOKV), *Duvenhage virus* (DUVV), *European bat lyssavirus,* types 1 and 2, and *Australian bat lyssavirus*. Another 4 lyssaviruses, all isolated from bats, have been assigned to the genus as putative species: Aravan virus, Khujand virus, Irkut virus, and West Caucasian bat virus (WCBV) (*3*).

RABV infection of bats is known in the Americas, but not in the Old World. Four lyssavirus species have been documented in Africa. Of these, RABV and MOKV have been isolated exclusively from terrestrial mammals, whereas LBV and DUVV are associated with bats and have been isolated only occasionally from other mammals (*4*). On the basis of this diversity and on the serologic cross-reactivity of MOKV with African non-lyssa rhabdoviruses, it has been hypothesized that Africa is the continent of the origin and initial evolution of members of the genus *Lyssavirus* (*5*). However, this hypothesis has been called into question by the isolation of WCBV in southeastern Europe. WCBV is the most divergent member of the genus *Lyssavirus* to date and has long genetic distances and lack of serologic cross-reactivity to other lyssaviruses (*6*,*7*). Our study objective was to enhance pathogen discovery for zoonotic agents in African bats, with particular focus on lyssavirus surveillance in Kenya.

## The Study

**Figure 1:**
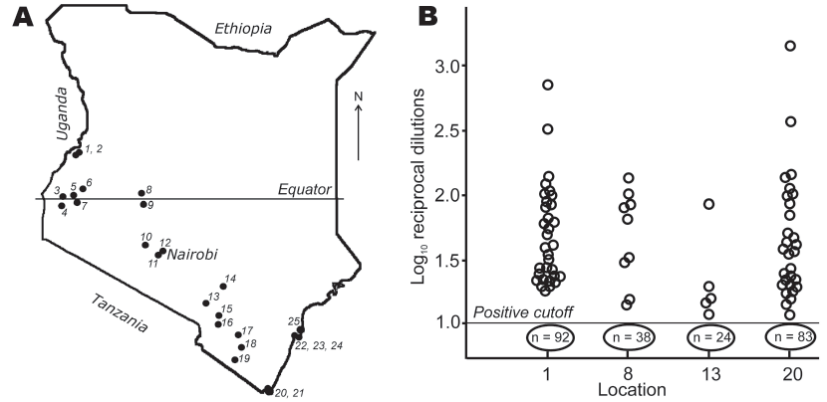
A) Map of Kenya showing locations of the bat collections, numbered in order of collection. B). Antibody titers to West Caucasian bat virus (WCBV) in *Miniopterus* bats from 4 of the locations. A modified rapid fluorescent focus inhibition test for WCBV-neutralizing antibodies was used. Bats with 50% end-point neutralizing titers >1 log_10_ were considered seropositive. Numbers of negative bats for each location are circled below the cutoff line.

During 2006–2007, bats of at least 30 species were collected from 25 locations in Kenya (Figure 1, panel **A**; Appendix Table). The sample numbers and collection protocol were approved by the National Museum of Kenya and the Kenya Wildlife Service. Of the 1,221 bats collected, only 12 were sick or dead; the others appeared healthy. Captured animals were anesthetized by an intramuscular injection of ketamine hydrochloride (0.05–0.1 mg/g) and euthanized under sedation in compliance with a field protocol approved by the Animal Institute Care and Use Committee of the Centers for Disease Control and Prevention. Bat size, sex, and species were identified. When phenotypic species determination was not possible, DNA specimens were submitted for identification to the University of Guelph (Ontario, Canada), where partial sequences of the cytochrome oxidase gene were generated and compared with those available from the Barcode of Life Data Systems (www.boldsystems.org).

The brain and other organs of bats were collected into sterile plastic tubes. Oral swabs were collected and placed in tubes containing Minimum Essential Medium (MEM-10, Invitrogen, Grand Island, NY, USA) for virus isolation or in TRIzol (Invitrogen, Carlsbad, CA, USA) for RNA extraction. Serum was separated from the blood clot by centrifugation. All samples were transported on dry ice and stored at –80^o^C.

The brains (n = 1,182) were subjected to the direct fluorescent antibody test for lyssavirus antigen (*8*). In addition, the 277 brains that were collected in 2006 were homogenized and tested for virus isolation by the intracerebral mouse inoculation test as described elsewhere (*9*). Virus isolation was attempted for only a subset of brain samples (n = 210) from the specimens collected during 2007.

Total RNA was extracted from the oral swabs (n = 931) and subjected to nested reverse transcription–PCR, as described previously (*10*). We used primers designed for the nucleoprotein genes of LBV, MOKV, and WCBV.

The virus neutralizing antibodies in bat serum samples were determined by a modification of the rapid fluorescent focus inhibition test. We used 4-well (6-mm) Teflon-coated glass slides (Cel-Line, Erie Scientific, Portsmouth, NH, USA) as described elsewhere (*10*). Previous tests for RABV neutralizing antibodies have demonstrated that results of this micromethod are comparable to those of the classical test in chamber slides. The neutralizing activity of each serum sample was determined against LBV, MOKV, DUVV, RABV, and WCBV. All samples were initially screened in dilutions of 1:10 and 1:25. Samples that showed reduced fluorescence or no fluorescence were subjected to additional titration in dilutions of 1:10 to 1:1,250. The 50% end-point neutralizing titers were calculated by the method of Reed and Muench (*11*). The samples that had a 50% end-point neutralizing titer >1 log_10_ were considered positive.

**Figure 2:**
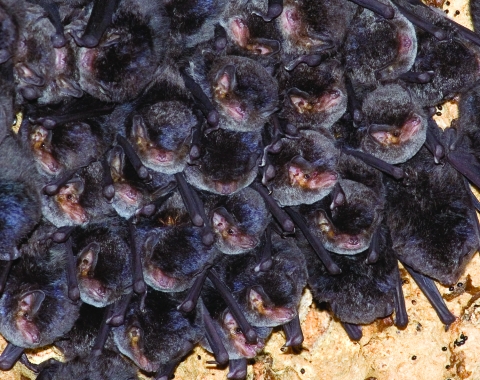
Colony of *Miniopterus minor* bats in cave.

Circulation of LBV was detected in fruit bats *Eidolon helvum* and *Rousettus aegyptiacus* as described previously (*10*). No other viruses were isolated during the study, and no lyssavirus RNA was identified in oral swabs. However, virus-neutralizing activity against WCBV was detected in serum of *Miniopterus* insectivorous bats (Figure 2) from 4 of the 5 locations where these species were collected (Appendix Table). Among 76 serum samples with WCBV-neutralizing activity (Figure 1, panel B), only 1 sample additionally neutralized DUVV, but no cross-neutralization to other lyssaviruses was detected. This observation supported specificity of the reaction and reliability of the selected cutoff threshold. The seroprevalence varied by roosts, 17% to 26% (95% confidence interval 17–27). In general, seroprevalence among females (n = 201; seroprevalence 26%) was greater than that among males (n = 112; seroprevalence 19%). Although statistically insignificant (χ^2^ = 2.38; p = 0.12), this difference was consistent across locations 1, 13, and 20. Only females were available from location 8. At all locations, *Miniopterus* bats shared caves with other species of insectivorous and fruit bats. However, no serologic activity against WCBV was detected in these other species. Of note, serum from fruit bats *R. aegyptiacus* that shared caves with *Miniopterus* bats neutralized LBV but not WCBV. Conversely, serum from *Miniopterus* bats neutralized WCBV but not LBV. This finding suggests that bats of different species, even those roosting in the same caves, do not readily expose each other to lyssaviruses.

## Conclusions

We found WCBV-neutralizing antibodies in bats in Africa. Because limited serologic cross-reactivity between lyssaviruses and other rhabdoviruses has been demonstrated (*12*), the WCBV seroprevalence we detected may have been caused by some other serologically related virus. However, to date no other agent that could cross-neutralize WCBV is known (*7*).

We cannot explain why 1 WCBV-neutralizing sample additionally neutralized DUVV. This finding could indicate nonspecific virus inhibition, or it could be evidence of coexposure of the bat to several lyssaviruses. Our inability to isolate viruses in this study is not surprising because lyssavirus prevalence in bat populations is usually low (<1%), even when seroprevalence is as high as 40%–70% (*10*,*13*,*14*). Indeed, the seroprevalence may reflect past exposures and peripheral virus activity rather than survival after clinical lyssavirus infection.

WCBV was first isolated in 2002 in southeastern Europe from *Miniopterus schreibersii* (*6*) bats, and only 1 isolate is available to date. Seroprevalence to this virus in African *Miniopterus* bats is intriguing. Perhaps WCBV and similar viruses are specifically associated with *Miniopterus* bats and distributed quite broadly. *Miniopterus* bats are common in the tropics and subtropics of the Old World (*15*). They segregate into large colonies in caves. For example, in Kenya, *Miniopterus* colonies consisted of thousands of bats. Many of these caves are regularly visited by local residents and by tourists. Although no data exist for WCBV pathogenicity in humans, the absence of reliable vaccine protection against this virus and the ability of WCBV to cause fatal encephalitis in animal models (*7*) suggest the need for improved surveillance and public education to avoid exposure to bats.

## Supplementary Material

Appendix Table Lyssavirus and WCBV-neutralizing antibody detection in bat tissues, Kenya, 2006-2007*
