# Supplementary material for: Possible Emergence of West Caucasian Bat Virus in Africa
Source: Emerg Infect Dis. 2008 Dec;14(12):1887–9. doi: 10.3201/eid1412.080750 (PMC2634633; doi:10.3201/eid1412.080750)
Supplement: Appendix Table — Lyssavirus and WCBV-neutralizing antibody detection in bat tissues, Kenya, 2006-2007* [file 08-0750_appT-s1.pdf]

Publisher: CDC; Journal: Emerging Infectious Diseases
Article Type: Dispatch; Volume: 14; Issue: 12; Year: 2008; Article ID: 08-0750
DOI: 10.3201/eid1412.080750; TOC Head: Dispatch

Appendix Table. Lyssavirus and WCBV-neutralizing antibody detection in bat tissues, Kenya, 2006–2007*

Location	Species	Tissue samples, no. positive/no. tested		
		Brains†	Oral swab‡	Serum§
1	*Miniopterus* spp.	0/155	0/110	33/125
	Rhinolophus spp.	0/31	0/24	0/16
	Rousettus aegyptiacus	0/76	0/75	0/76
2	*Hipposideros ruber*	0/4	–	0/3
	Rhinolophus spp.	0/1	0/1	0/1
	Rousettus aegyptiacus	0/56	0/54	0/54
3	*Chaerophon pumilus*	0/8	0/8	0/8
	Eidolon helvum	0/18	0/17	0/18
	Epomophorus labiatus	0/6	0/6	0/5
4	*Chaerophon pumilus*	0/3	–	0/4
	Chaerophon spp.	0/8	–	0/8
5	*Eidolon helvum*	0/86	0/86	0/79
6	*Hipposideros ruber*	0/2	–	0/2
	Lissonycteris angolensis	0/10	–	0/11
	Miniopterus inflatus	0/12	–	0/11
7	*Chaerophon* spp.	0/17	–	0/16
	Neoromicia tenuipinnis	0/4	–	0/4
8	*Miniopterus* spp.	0/47	0/50	9/47
	Rhinolophus hildebrandtii	0/1	0/1	0/1
	Rhinolophus landeri	0/6	0/6	0/4
	Rhinolophus spp.	0/1	0/1	0/1
9	*Rhinolophus landeri*	0/9	0/8	0/6
10	*Otomops martiensseni*	0/19	–	0/19
11	*Pipistrellus* spp.	0/1	–	0/1
	Rhinolophus spp.	0/6	–	0/6
12	*Epomophorus wahlbergi*	0/3	–	0/3
	Epomophorus labiatus	0/1	–	–
13	*Miniopterus africanus*	0/29	0/31	5/29
	Rhinolophus huldebrandtii	0/21	0/16	0/19
	Rhinolophus landeri	0/2	0/2	0/2
	Rhinolophus spp.	0/4	0/4	0/4
14	*Pipistrellus* spp.	0/1	0/1	0/1
15	*Chaerophon pumilus*	0/13	0/13	0/12
	Epomophorus wahlbergi	0/2	0/2	0/2
	Nycteris spp.	0/1	–	0/1
	Neoromicia spp.	0/2	0/2	0/2
16	*Coleura afra*	0/12	0/12	0/13
	Rhinolophus landeri	0/1	0/1	0/1
	Rhinolophus spp.	0/15	0/13	0/14
17	*Epomophorus wahlbergi*	0/7	0/8	0/8
	Nycteris spp.	0/1	0/1	0/1
	Pipistrellus spp.	0/2	0/2	0/1
18	*Chaerophon pumilus*	0/6	–	0/6
	Coleura afra	0/18	–	0/2
	Taphozous spp.	0/2	–	0/2
19	*Cardioderma cor*	0/12	–	0/11
	Species unidentified	0/4	–	0/4
20	*Coleura afra*	0/5	0/2	0/1
	Hipposideros commersoni	0/6	0/4	0/6
	Miniopterus minor	0/134	0/120	29/112
	Nycteris hispida	0/4	0/4	0/4
	Rhinolophus spp.	0/1	–	–
	Rousettus aegyptiacus	0/107	0/106	0/93
	Triaenops persicus	0/16	0/18	0/12
21	*Coleura afra*	0/1	–	0/1
	Hipposideros commersoni	0/10	–	0/10
	Rhinolophus spp.	0/2	–	0/2
	Taphozous hildegarde	0/3	–	0/2
22	*Cardioderma cor*	0/14	–	0/13
23	*Pipistrellus* spp.	0/1	–	0/1
	Rousettus aegyptiacus	0/106	0/117	0/116
	Scotophilus spp.	0/1	–	0/1
24	*Eidolon helvum*	0/5	0/5	0/5
25	*Chaerophon* spp.	0/20	–	0/19
Total		0/1,182	0/931	76/1,062

*WCBV, West Caucasian bat virus.

†Both the direct fluorescent antibody test and the mouse inoculation test were used for 487 samples; the direct fluorescent antibody test was used only for the remaining 695 samples.
‡Oral swabs tested by nested reverse transcription–PCR.
§Tested by rapid fluorescent focus inhibition test for WCBV–neutralizing antibody.
